# Seasonal variation in objectively assessed physical activity among children and adolescents in Norway: a cross-sectional study

**DOI:** 10.1186/1479-5868-6-36

**Published:** 2009-06-29

**Authors:** Elin Kolle, Jostein Steene-Johannessen, Lars B Andersen, Sigmund A Anderssen

**Affiliations:** 1 Department of Sports Medicine, Norwegian School of Sport Sciences, P.O. Box 4014 Ullevål Stadion, N-0806 Oslo, Norway; 2 Research in Childhood Health, University of Southern Denmark, Odense, Denmark

## Abstract

**Background:**

The literature on seasonality in children and youth's physical activity participation is inconsistent. The aims of this study were to: 1) compare physical activity across seasons and describe activity patterns within seasons, and 2) to determine compliance with current physical activity recommendations across seasons among 9- and 15-year-olds living in a climatically diverse country.

**Methods:**

Participants were 2,299 9- and 15-year-olds from all regions in Norway. Physical activity was assessed using the Actigraph accelerometer for 4 consecutive days. Physical activity data were collected during winter, spring and fall. General linear models were used to study the associations between physical activity and season.

**Results:**

Nine-year-old children had significantly higher mean physical activity levels in spring than in winter and fall. In the two latter seasons, physical activity levels were especially low after school hours and on weekends. Logistic regression models demonstrated that 9-year-olds had 3.3 times (95% Confidence Interval (CI): 2.08, 5.18) higher odds of meeting recommended levels of physical activity in spring than in winter. No associations were found between mean physical activity level and season among the 15-year-olds. However, the adolescents also had higher odds (OR = 1.56; 95% CI: 1.05, 2.32) of meeting the physical activity recommendations in spring than in winter.

**Conclusion:**

In a large population-based sample, we observed substantial seasonal differences in physical activity among 9-year-olds, and the activity pattern varied across the seasons. The results emphasize the need to take season into account when developing physical activity interventions for children. Season appears to have less influence on adolescent's physical activity; interventions for increasing physical activity in this group could therefore be implemented throughout the year.

## Background

Despite the immediate and likely long-term benefits of physical activity in childhood [1,2], many children and particularly adolescents, fail to meet the recommended 60 minutes of moderate-to-vigorous physical activity (MVPA) daily [3-5]. Given this situation, several interventions have been developed to promote physical activity participation in the young population. Successful and effective interventions depend on a thorough understanding of the determinants of physical activity [6]. Recently, studies have sought to understand the role of environmental variables [7] such as neighbourhood, school, and community characteristics, in physical activity participation. As physical activity is performed in specific physical settings, it is believed that environmental conditions exert either a facilitating or constraining influence on physical activity [8].

Seasonality has received little attention as a potential environmental determinant of physical activity. In temperate and polar regions four seasons are generally recognized (winter, spring, summer and fall). Temperatures, precipitation, and day length may vary substantially across seasons, and such attributes might affect physical activity participation. The literature on seasonality in children and youth's physical activity participation is inconsistent. While some studies have shown that season has an impact on physical activity [9-14], other studies fail to do so [14,15]. Furthermore, few studies have described physical activity patterns during different seasons. The use of accelerometers to assess physical activity provides an opportunity to improve the understanding about the duration, intensity, and frequency of activity. Such knowledge is useful for intervention and health promotion planning, as it might be able to identify seasons that can be targeted for promotion of physical activity.

Norway comprises the western part of Scandinavia and stretches over 2,500 km. The country is climatically diverse, and due to it's high latitude (latitude range: 57° N to 72° N, longitude: 10° E), there are large seasonal variations in daylight. It is possible that in countries where seasonal variations are large, daily physical activity may be more influenced by seasons than in countries where seasonal variations are smaller. Thus, the aims of this study were to: 1) compare physical activity across seasons and describe activity patterns within seasons, and 2) to determine compliance with current physical activity recommendations across seasons among 9- and 15-year-olds living in Norway.

## Methods

This is a national, cross-sectional examination of randomly selected 9- and 15-year-old children and adolescents (fourth and tenth grade) in Norway. Statistics Norway selected the cohorts by cluster sampling, with schools as the primary unit. When a school consented to participate, all children in fourth or tenth grade were invited to participate in the study. We recruited subjects from 40 elementary schools and 23 high schools representing all regions in Norway. Of 2,818 invited participants, 2,299 agreed to participate, giving a participation rate of 89% among the 9-year-olds and 74% among the 15-year olds. The study was approved by the Regional Committee for Medical Research Ethics and Norwegian Social Science Data Services. Each participant's parent or guardian provided written consent before he or she was included in the study.

### Measures

Height and weight were measured by standardized procedures and Body mass index (BMI) was calculated as weight (kg) divided by the height squared (m^2^).

### Physical activity

The uni-axial MTI Actigraph accelerometer (model 7164; Manufacturing Technology Inc., Fort Walton Beach, FL) was used to assess physical activity. This is an electronic motion sensor comprising a single plane (vertical) accelerometer. Movement in the vertical plane is detected as a combined function of the frequency and intensity of the movement. The Actigraph accelerometer has been validated in both children and adolescents against heart-rate telemetry [16], indirect calorimetry [17], observational techniques [18], and energy expenditure measured by doubly-labelled water [19].

The participants were visited at their school, and each child and adolescent was fitted with an accelerometer in an elastic belt around their waist, worn for 4 consecutive days (2 weekdays and 2 weekend days). Children and adolescents were asked to wear their accelerometers during waking hours and to take it off only for showering, bathing or water sports. The Actigraphs were initialized to start recording at 6 am on the day following distribution. An epoch time of 10 seconds was used. A SAS-based software program (SAS Institute Inc., Cary, North Carolina, USA) called CSA-analyzer (csa.svenssonsport.dk) was used to analyze accelerometer data. In the analysis, we excluded all night activity (between 12 am and 6 am), and ten or more minutes of consecutive zeros were regarded as periods in which the monitor was unworn, and these were deleted from each file. A newly published study (20) reported that the single-day intraclass correlation coefficient (ICC) for 600 minutes of assessment was 0.45, while the ICC for 480 minutes of assessment was 0.44. To avoid loosing statistical power, we chose to specify a valid day as 480 minutes. In the present study, data were considered valid if a child provided a minimum of 2 days of at least 480 minutes per day recorded. A total of 1,824 (79%) subjects provided valid physical activity recordings. Reasons for exclusion (N = 475) were failing to achieve at least two days of measurement (25%), not wearing the accelerometer (36%), and instrument malfunction (39%). Although a weekend day was not specified in order to fulfil validity criteria, 93% of the participants had at least one weekend day of recording.

The total amount of physical activity from the activity monitor was expressed as the average of total counts per minute of registered time (counts/min). We defined MVPA as all activity above 2,000 counts/min. This threshold corresponds to a walking pace of about 4 km/h in children (3 metabolic equivalents) [20,21] and has been applied in previous studies [22-24]. The proportion of children and adolescents who achieved the recommended 60 minutes of daily MVPA was established by dividing total time in MVPA (min) by the number of valid days of recording, giving an average (min/day) across the assessment period.

### Season

Data were collected from March 2005 through October 2006, with physical activity assessments throughout the year, except during summer vacation (July and August). Seasons were defined as periods which fluctuate by weather conditions, daylight hours and temperature, as: "Winter", 1 December – 28 February; "Spring", 1 March – 15 June; and "Fall", 1 September – 30 November. Note that assessments taken in June (summer) (N = 121) were categorized as "Spring".

### Statistical analysis

Data are presented as mean (SD) unless otherwise stated. To assess potential differences in activity levels between subjects with different numbers of assessment days, we calculated physical activity levels separately for subjects with 2, 3 and 4 days of valid activity recordings. No differences in mean physical activity were found between subjects with different number of assessment days (9-yrs: p = 0.085; 15-yrs: p = 0.201). Further, we did the seasonal analyses including only subjects with ≥3 days of valid physical activity assessment and the results did not alter our conclusions. To avoid the loss of statistical power we chose to include children with ≥2 days of valid physical activity recordings in the analyses. General linear models were used to study the associations between sex, age group, mean physical activity and season. We found no three-way interaction between sex, age group and season, however, the analyses revealed an interaction between season and age group (p = 0.001) and sex and age group (p = 0.03). Consequently the analyses were run separately for each sex and age group. Logistic regression analysis was applied to study the percentage meeting the physical activity recommendations in relation to sex, age group and season. As an interaction was found between sex and age group (p < 0.001) the analysis was run separately for each age group. The results are presented as adjusted odds ratios (ORs) with 95% CIs. All analyses were performed by using the Statistical Package for Social Sciences (SPSS, version 15.0).

## Results

Valid physical activity assessments were obtained from 1,127 9-year-olds (525 girls and 602 boys) and 697 15-year-olds (359 girls and 338 boys). The mean anthropometric data and mean physical activity data by sex and age group are shown in Table 1, which also shows the numbers of participants studied in each of the three seasons. In each age and sex group, there was no significant difference in height, mass or BMI (when adjusting for age) among the participants measured in the different seasons.

**Table 1:** Characteristics of the participants by sex and age group (*N *= 1824)

	**9-yrs**		**15-yrs**	
		
	**Girls**	**Boys**	**Girls**	**Boys**
N	525	602	359	338
Height (cm)	138.4 (7.0)	139.9 (6.2)	165.6 (6.5)	175.7 (7.1)
Weight (kg)	34.0 (7.1)	33.9 (6.3)	57.9 (8.7)	65.2 (12.8)
Body Mass Index (kg/m^2^)	17.6 (2.7)	17.2 (2.4)	21.1 (2.8)	21.1 (3.7)
Physical activity (PA)				
Days of PA assessment	3.6 (0.6)	3.7 (0.6)	3.4 (0.8)	3.3 (0.8)
Total PA^a ^(min/day)	759 (59)	769 (61)	779 (77)	778 (80)
Mean PA (counts/min)	693 (251)	796 (281)	487 (167)	542 (199)
MVPA (min/day)	76 (23)	95 (31)	62 (25)	68 (28)
Season, N (%)^b^				
Winter	74 (14)	88 (15)	110 (31)	115 (34)
Spring	248 (47)	304 (50)	83 (23)	92 (27)
Fall	203 (39)	210 (35)	166 (46)	131 (39)

Values are mean (SD) unless otherwise mentioned

PA. physical activity; MVPA, moderate-to-vigorous physical activity

^a^Total minutes of recorded physical activity each day

^b^The number (%) of participants studied in each season

Winter, 1 December – 28 February; Spring, 1 March – 15 June; Fall, 1 September – 30 November

Mean physical activity levels by sex, age group and season are displayed in Table 2. No significant differences in mean number of valid assessment days or mean number of valid physical activity recordings per day were detected between children assessed in different seasons (p > 0.05). Among 9-year-olds, there were significant seasonal differences in mean physical activity level. Both 9-year-old girls and boys were significantly more active during spring than during winter (girls: mean difference: 188 counts/min, 95% CI: 112, 265, p < 0.001; boys: mean difference 121 counts/min, 95% CI: 41, 201, p = 0.001) and fall (girls: mean difference: 112 counts/min, 95% CI: 57, 167, p < 0.001; boys: mean difference: 113 counts/min, 95% CI: 53, 172, p < 0.001). There were no seasonal differences in mean physical activity levels in the 15-year-old girls or boys.

**Table 2:** Mean (SD) physical activity level (counts/min) stratified by sex, age group and season

	**9-yrs**				**15-yrs**			
		
	**Girls**		**Boys**		**Girls**		**Boys**	
		
**Seasons**	**N**	**Mean (SD)**	**N**	**Mean (SD)**	**N**	**Mean (SD)**	**N**	**Mean (SD)**
Winter	74	575 (141)	88	732 (213)	110	460 (157)	115	520 (180)
Spring	248	763 (294)	304	853 (322)	83	503 (185)	92	580 (204)
Fall	203	651 (195)	210	741 (219)	166	497 (164)	131	535 (207)

**Figure 1:**
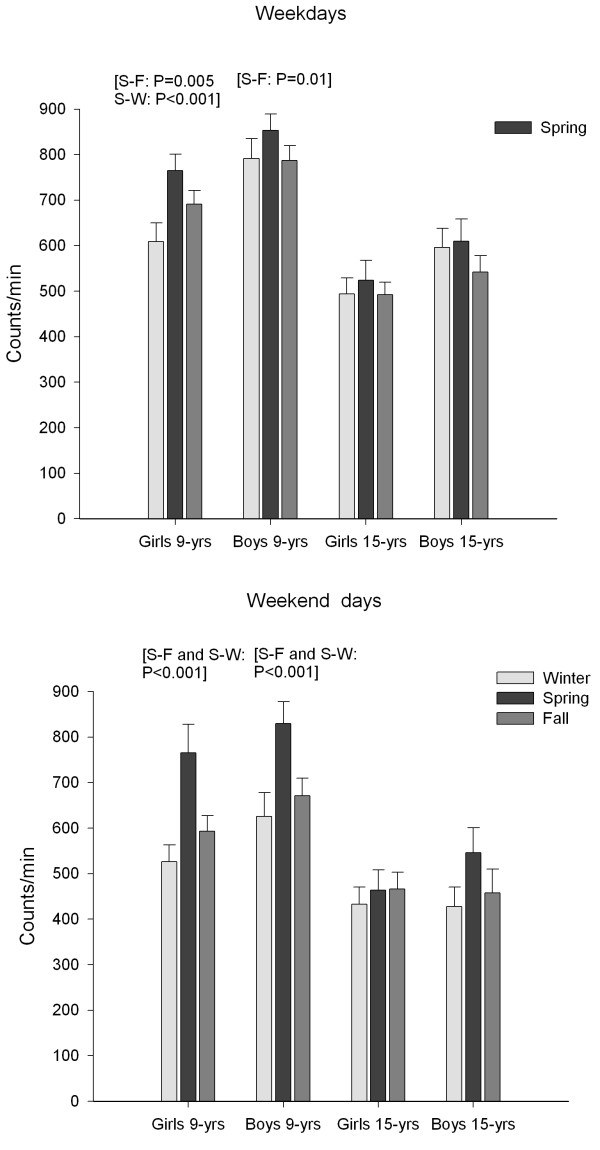
**Weekday and weekend physical activity**. Mean (95% CI) physical activity level (counts/min) during weekdays (top) and weekend days (bottom) stratified by sex, age group and season. W, Winter; S, Spring; F, Fall.

Among 9-year-olds, the seasonal variation in mean physical activity level was particularly large during weekends (Figure 1). Among girls, the spring-winter difference in weekend physical activity was 240 counts/min (95% CI: 120, 361; p < 0.001) while the spring-fall difference was 174 counts/min (95% CI: 87, 260; p < 0.001). Among boys, the spring-winter difference in weekend physical activity was 204 counts/min (95% CI: 99, 309; p < 0.001) while the spring-fall difference was 159 counts/min (95% CI: 82, 237; p < 0.001). Smaller, but statistically significant differences were also found in 9-year-olds' weekday physical activity. Among girls, the spring-winter difference in weekday physical activity was 158 counts/min (95% CI: 80, 236; p < 0.001) while the spring-fall difference was 73 counts/min (95% CI: 17, 129; p = 0.005). Among boys, the only significant difference in weekday physical activity was found between spring and fall where the difference was 74 counts/min (95% CI: 12, 135; p = 0.01). The results revealed no significant association between season and mean physical activity level during weekdays or weekends among the 15-year-old girls and boys.

**Figure 2:**
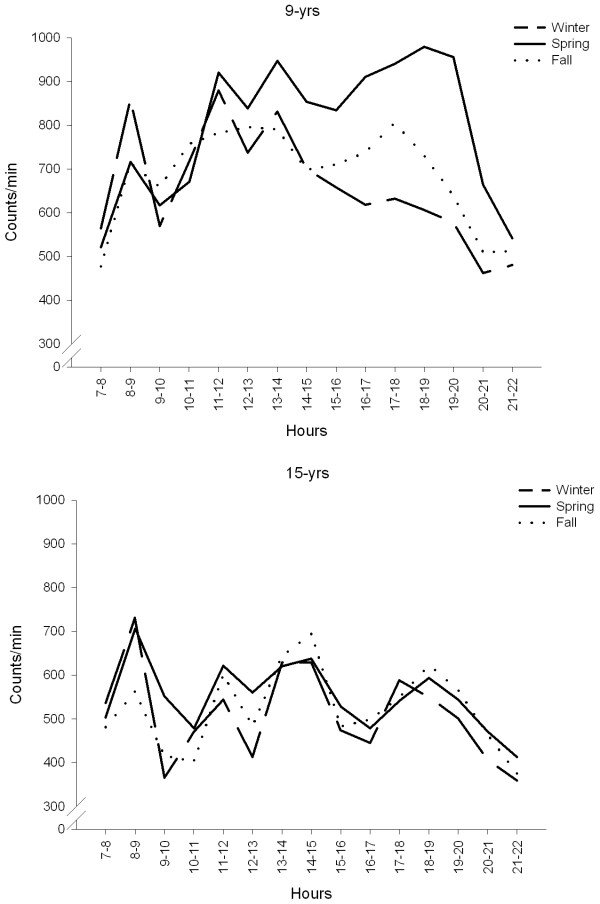
**Daily physical activity patterns of 9- and 15-year-olds during the three seasons**. Plotted values are mean physical activity level (counts/min).

Figure 2 shows the daily activity patterns of 9- and 15-year-olds during the three seasons. For the 9-year-olds, marked differences in activity patterns were observed between the three seasons, particularly in the period between end of school and bedtime. Activity levels were higher during spring and lower physical activity during winter. For the 15-year-olds, the daily activity patterns during the three seasons were remarkably similar. The activity pattern was characterised by several peaks throughout the day, but none of the seasons was characterised by especially low or especially high physical activity levels.

Among 9-year-olds, the odds of meeting recommended levels of physical activity were higher among boys than among girls (Table 3). No difference in odds was observed by sex among the 15-year-olds. Both 9- and 15-year-olds had higher odds of meeting physical activity recommendations during spring than during winter.

**Table 3:** Odds ratios (and 95% CI) for meeting physical activity recommendations by sex and age goup

	**Odds Ratio (95% Confidence Interval)**	
	
	**9-yrs**	**15-yrs**
Sex		
Girls	1.00	1.00
Boys	3.19 (2.26, 4.49)*	1.19 (0.88, 1.61)
Season		
Winter	1.00	1.00
Spring	3.28 (2.08, 5.18)*	1.56 (1.05, 2.32)**
Fall	1.44 (0.93, 2.22)	1.28 (0.90, 1.81)

*P < 0.001, **P = 0.03

## Discussion

Objective assessment of physical activity revealed seasonal differences in physical activity in 9-year-old children living in a climatically diverse country. Norwegian 9-year-olds had higher physical activity levels in spring than in fall and winter. In the two latter seasons, activity levels were particularly low after school hours and on weekends. No seasonal differences in mean physical activity were observed among the 15-year-olds. However, both 9- and 15-year-olds had higher odds of meeting recommended levels of physical activity during spring than during winter.

Associations between season and children's daily physical activity have been reported in several other countries. For example, in US pre-schoolers [10] an association has been reported between season and physical activity assessed by observation, activity was consistently higher outdoors than indoors, and outdoor activity was lower during the summer months. In contrast, a Scottish study [11] found that physical activity (assessed by accelerometers) was highest in summer and lowest in spring. As was the case in this study, several others have shown that younger children's physical activity levels are higher in spring than at other times of the year. For example, a study with 7-year-old children living in Vermont and Alabama, reported higher total energy expenditure measured by doubly-labelled water in spring than in fall [25], and a study in the southern US [26], which used pedometers to assess physical activity, also found that activity levels were higher in spring than in winter among first through fifth grade students. Similar results were reported in a pedometer study with 8–10-year-old boys in the UK [13]. Higher activity levels (assessed by accelerometers) were also reported during spring than during summer, fall and winter in Danish 8–10-year-old girls and boys [14]. In general, it seems clear that seasons have some effect on children's activity level; five out of six studies reported higher activity levels in spring [13,14,25,26] or summer [11], while the remaining study reported lowest activity level in summer [10]. The low summer activity level in the latter study is probably explained by the extreme heat experienced in Texas during summer.

Research studying the impact of season on adolescents' (individuals aged 11–17-years) physical activity level has shown conflicting results. Studies conducted among 15-year-olds in Denmark [14] and the United States [15] found no association between season and physical activity assessed by accelerometers and 7-day recall questionnaire, respectively. Conversely, a study including 10–17-year-old Portuguese adolescents indicated that physical activity level assessed by questionnaire was significantly higher in summer/spring than in fall/winter [27]. Moreover, a study including 11–12-year-olds in the UK reported that physical activity assessed by accelerometer was highest in summer and lowest in winter [12]. Measurement error, different sample characteristic, different geographic regions, and different analysis strategies all increase the likelihood of inconsistent findings across studies.

The reason for reduced physical activity level during fall and winter remains unclear, however, weather and daylight availability might be the key determinants for low physical activity levels during the cold seasons. In Oslo (the capital), the hours of daylight differ from approximately 6 hours in December to 19 hours in June, while the areas above the Arctic Circle experience both months with no daylight (winter) and months where the sun never sets (summer). In addition, the months of fall and winter are often characterized by periods of continuous poor and harsh weather (rain, snow and wind), which have shown to be strong deterrents to daily physical activity [28]. A combination of low daylight availability and continuous harsh weather might lead to children spending more time indoors. As time spent outdoors is a significant predictor of children's physical activity [6,10], the consequence might be reduced physical activity level during fall and winter, and lower odds of meeting recommended levels of physical activity.

Our results suggest the after-school time period as an opportunity to promote physical activity. After-school programs are optimal for enhancing physical activity because of the capacity and infrastructure to reach large numbers of children, and both organized and unorganized activities can be promoted. One study from the US [29] has shown that children in grade 3 through 6 accumulate significant amounts of MVPA while attending after-school programs. Physical activity levels were higher during free-play sessions than in organized or structured activity. In Norway, after-school programs could use indoor facilities, sport halls, and swimming pools to facilitate children's natural inclination to move and play during fall and winter when playing outdoors is impractical. However, this would require qualified teachers and leaders with skills in physical activity instruction and programming. After school activities should be enjoyable [30] and affordable so that children from all socioeconomic groups can participate. Parental support, which is known to be an important determinant of children's physical activity would also be crucial [31]. Through encouragement, provision of transport, participation with the child and observation of the child being active, parents can have a strong influence on children's physical activity [32,33]. It is therefore essential to increase parents' awareness of the importance of adequate physical activity, so they can provide opportunities for physical activity at times of the year when the levels are low.

It appears that seasonality has limited effect on physical activity behaviour during adolescence. This period is characterized by a decrease in physical activity [34], which is caused both by a decline in both organized and non-organized activities. As children go through adolescence there is less free play outdoors, and physical activity mainly consists of participation in organized leisure-time activities, for example in sport clubs. It is therefore plausible that 15-year-olds' physical activity is less influenced by fluctuation in daylight and weather, as most organized activities take place all year around. Since seasonal variations are less important for physical activity participation in adolescence, interventions to increase physical activity should be targeted all year round. A newly published Norwegian study [35] showed that 54% of 15-year-old girls and 62% of 15-year-old boys were members of a sports club. One solution to increase 15-year-olds' physical activity levels might be to provide more organized activities.

A major strength of this study was the use of objective monitoring to assess both the quantity and intensity of physical activity. This is important as children lack the cognitive ability to accurately recall details of their physical activity patterns [36]. Also, the use of accelerometers are known to compare favourably with other similar instruments such as pedometers [37]. Furthermore, our study included a large random selected study sample and the participation rate was high.

A limitation of the study was the use of a cross-sectional design and thus the lack of physical activity assessments at multiple time points. This was, however, not possible due to the time- and labour-consuming nature of conducting multiple physical activity assessments. Further, we did not collect data on temperature, precipitation, amount of daylight or time spent outdoors, which may be important because the weather and daylight hours can differ significantly across the seasons.

## Conclusion

Norway is climatically diverse country with large seasonal variations in temperature, precipitation and hours of daylight. In a large population-based sample, we observed substantial differences in physical activity levels and patterns among 9-year-olds across the seasons. Our results emphasize the importance of taking season into account when promoting physical activity in children. When promoting physical activity among adolescents, season is of less importance and interventions aiming to increase physical activity should be implemented throughout the year. Finally, this study emphasizes the need to take season into consideration when conducting prevalence studies of physical activity in children.

## List of abbreviations

BMI: Body Mass Index; CI: Confidence Interval; MVPA: Moderate-to-Vigorous Physical Activity.

## Competing interests

The authors declare that they have none competing interests.

## Authors' contributions

EK was active in the planning of the study and coordinated the data collection, did the conception and design, analysed and interpreted the data and drafted the manuscript. JSJ was active in the planning of the study and coordinated the data collection, was involved in the conception and design, discussed the analysis and interpretation of the data and reviewed the manuscript critically. LBA was active in the planning of the study, was involved in the conception and design of the article, contributed particularly in the statistical analyses and interpretation of the data, and reviewed the article critically. SAA was project manager of the study, was active in planning of the study, was involved in the conception and design of the article, discussed the analysis and interpretation of the data, and reviewed the manuscript critically.
